# How do established developmental risk-factors for schizophrenia change the way the brain develops?

**DOI:** 10.1038/s41398-021-01273-2

**Published:** 2021-03-08

**Authors:** Darryl W. Eyles

**Affiliations:** 1 Queensland Brain Institute, University of Queensland, Brisbane, 4072 QLD Australia; 2 Queensland Centre for Mental Health Research, The Park Centre for Mental Health, Wacol, 4076 QLD Australia

**Keywords:** Neuroscience, Molecular neuroscience

## Abstract

The recognition that schizophrenia is a disorder of neurodevelopment is widely accepted. The original hypothesis was coined more than 30 years ago and the wealth of supportive epidemiologically data continues to grow. A number of proposals have been put forward to suggest how adverse early exposures in utero alter the way the adult brain functions, eventually producing the symptoms of schizophrenia. This of course is extremely difficult to study in developing human brains, so the bulk of what we know comes from animal models of such exposures. In this review, I will summarise the more salient features of how the major epidemiologically validated exposures change the way the brain is formed leading to abnormal function in ways that are informative for schizophrenia symptomology. Surprisingly few studies have examined brain ontogeny from embryo to adult in such models. However, where there is longitudinal data, various convergent mechanisms are beginning to emerge involving stress and immune pathways. There is also a surprisingly consistent alteration in how very early dopamine neurons develop in these models. Understanding how disparate epidemiologically-validated exposures may produce similar developmental brain abnormalities may unlock convergent early disease-related pathways/processes.

## Introduction

Schizophrenia is a devastating disorder leading to psychosis, severe impairments in cognition and social interaction. Like all complex psychiatric conditions, its aetiology remains unknown. Without the benefit of a clear pathology, treatments have been limited largely to symptom control. Whilst antipsychotic drugs can largely treat the psychotic symptoms in most patients the so-called negative and cognitive symptoms remain and the use of antipsychotic drugs comes with a substantial side-effect burden. Clearly, a better aetiological understanding is needed to develop more effective therapies. One longstanding hypothesis regarding the origins of schizophrenia is that it is a disorder of brain development^1,2^. The epidemiological landscape of schizophrenia suggests exposure to a wide variety of environmental risk factors (RF) during either foetal or early postnatal life leads to a modest increase in schizophrenia incidence^3^. There are also well-described exposures during late adolescence such as exposures to drugs of abuse such as cannabis^4^ or to various social stressors that also increase the risk of developing schizophrenia. Whilst such late adolescent exposures are an important component contributing to overall disease risk, they are not the focus of this review.

The purpose of this review is to describe how adverse developmental environments collectively initially launch the developing brain on a journey towards schizophrenia. Here I will focus on developmental RFs that have been robustly replicated by multiple epidemiological studies. Such RFs include maternal infection/inflammation; obstetric complications focussing on pre- and perinatal hypoxia; prenatal stress and prenatal nutrition. These RFs also have existing animal models in which the effects on foetal/developing brains are periodically reported. In this review, I will summarise the convergent cellular and molecular data from these preclinical models.

Of course, the developmental environment does not act in a genetic vacuum. Prior to the current genome-wide association study (GWAS) era, gene × environment interactions were examined by studying highly penetrant single nucleotide variants and their interaction with adverse environments. One example of such an approach is that of variants in the catechol‐O‐methyltransferase gene and cannabis use. Unfortunately, such interactions in patients frequently proved to have either no interaction or be additive in effect^5^. Today GWAS studies across multiple psychiatric conditions reveal a polygenetic genetic landscape with risk conferred by numerous variants of very small effect size. In order to describe the actual genetic variability within patients at a single nucleotide variant level, researchers have now compiled risk scores encompassing this variance, the so-called polygene risk score (PGRS). Although only accounting for about 7% of the total genetic variance of the disease^6^, the PGRS for schizophrenia has become widely used largely because of its modest diagnostic predictive power. This field is rapidly moving with attempts now to integrate the environment in which such genetic variance acts by creating genome-wide environmental interaction studies (GWEIS)^7,8^.

**Fig. 1:**
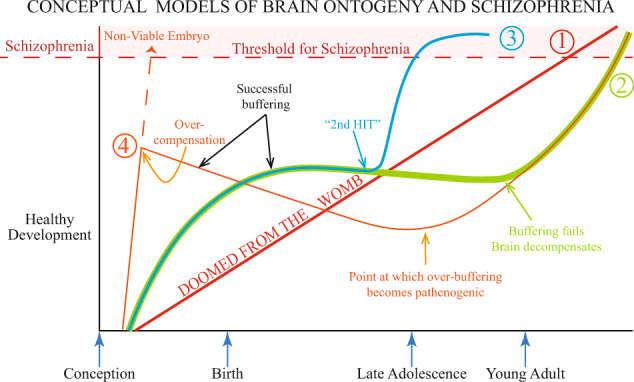
Conceptual models of brain ontogeny and schizophrenia. Depicted are four scenarios regarding how abnormal brain development could be a causal factor in patients developing schizophrenia. The four options are described in detail in the text. Options 1–3 have all been outlined previously by others. Option 4 is highly speculative and based largely on observations on dopamine ontogeny in animal models of developmental risk factors for schizophrenia. Thereof course may be many other conceptual models for how abnormal brain development alters brain ontogeny contributing to schizophrenia.

In considering the trajectory of how an adverse developmental environment may affect brain ontogeny in the progression towards disease there are a number of conceivable possibilities. A summary of some of the many possibilities is presented in (Fig. 1).

1. Developmental RFs cause an early static lesion that progresses with an inevitable transition to schizophrenia. This is the so-called “doomed from the womb “ scenario.
2. Developmental RFs induce an early lesion/pathology/circuit dysfunction but this is initially successfully buffered. However, such a situation becomes unmasked when those distributed buffering/compensating circuits become “hard-wired” in young adults and are perhaps no longer capable of buffering the developmental insult.
3. RF-induced early lesion/pathology/circuit dysfunction is successfully buffered such that the individual would never stray across the threshold for the disease until exposed to a further post-adolescent insult in which another circuitry becomes engaged and the original buffering mechanisms are proved to be inadequate. This is the so-called “2 hit model”.
4. Another and far more speculative model is proposed based largely on observations in preclinical models. It is possible that a severe, say life-threatening embryonic alteration/lesion occurs that unless immediately buffered/compensated would be incompatible with life. In this model, it is proposed that such extreme buffering/overcompensation eventually creates a pathogenic process that itself cannot be successfully buffered. Later we outline our findings in developing dopamine systems in animals that provide the basis for this last conceptual model.

Obviously, there may be overlapping competing/synergistic mechanisms across any such model^7^. In this review, we will examine the potential various pathogenic/protective mechanisms operating during pre- and postnatal brain development that may influence the course of the disease. Because of the invasive nature of such studies and the long time frame involved in human pre- and postnatal brain development, researchers turn to animal models. Historically, developmental models of relevance to schizophrenia have been used to assess drug efficacy, their so-called “*predictive validity*”. Less common are investigations of the initial alterations induced by such models on foetal and postnatal brain ontogeny. In this review, I hope to convince the reader that focusing on how such RFs change the very early maturation of cells/circuits in developing brains, represents a means to discover convergent, possibly causal, pathways to disease.

## What are the major developmental RFs for schizophrenia?

For more than 35 years we have known that complications during pregnancy can produce long-lasting impediments in neurological function in offspring^9^. Obstetric complications represent the most enduring developmental RF in schizophrenia epidemiology^10^. However “obstetric complications” can be used to cover virtually any adverse event during pregnancy. The obstetric complications most commonly linked with schizophrenia are acute hypoxia due to extended labour or longer-term hypoxia, i.e., due to maternal smoking or pre-eclampsia. In general, hypoxia-related events mediated via reactive oxygen species, inflammatory responses or diminished nutrient supply are considered causal mechanisms. For a summary of the clinical epidemiology see the review by Cannon et al.^10^.

Prenatal infection has also long been linked with an increased incidence of schizophrenia. Retrospective studies conducted over the last 100 years have shown children born during major infectious epidemics to agents such as influenza measles, polio, diphtheria have in general (but not always) shown increased rates of schizophrenia^11^. More recent work has clarified links between specific viral or bacterial exposures and disease outcome. It is of interest to speculate whether the maternal infectious agent itself actually reaches the developing brain to initiate local infections or whether inflammatory cytokine molecules produced by the mother’s immune response mediate the pathogenic mechanism/s? This latter proposal is supported by simply elevating maternal temperature in the absence of any infectious agent to mimic fever which itself produces adverse outcomes for the developing brain^12^. There may even be some antibody-mediated or auto-immune process initiated^13^.

Maternal stress is also another well-validated RF for schizophrenia in offspring^14^. Adverse events during pregnancy such as depression, unwanted pregnancy, death of a partner, and exposure to war and disasters, are associated with an increased incidence of schizophrenia in offspring^15^. A persistent alteration in glucocorticoid production via the maternal hypothalamic pituitary axis is believed causal. At a molecular level, we have known for more than 25 years that high or low levels of glucocorticoids differentially shape developing brain cells^16^.

Finally, maternal nutrition has long been associated with schizophrenia risk. Reassessment of historical famines highlighted the role of pre- and peri-natal nutrition. For instance, those conceived during the height of the Dutch famine during the second world war exhibited a two-fold increase in schizophrenia during adulthood^17^. This finding was replicated in two studies examining those individuals conceived during the 1959–1961 Chinese famine^18,19^. This risk was found to be higher in rural areas, where the severity of famine was greater^19^. The famine studies described above were not able to identify whether schizophrenia risk was mediated by a deficiency of a specific nutrient, or via general effects of malnutrition. In recent decades, numerous epidemiological studies and intervention trials have attempted to elucidate the role of individual nutritional candidates, in the hope of identifying preventive or treatment interventions. To date, the maternal nutrient deficiencies most closely linked with schizophrenia include maternal protein, polyunsaturated fatty acids, folate, iron and vitamin D^20^.

## Is there a specific window of vulnerability during pregnancy that increases schizophrenia risk?

In terms of brain development, pregnancy has an orderly sequence of definable stages. Therefore, an important factor to consider is whether there are specific stages of pregnancy that are more vulnerable? In respect to obstetric complications, most studies have focused on adverse events during labour. Far fewer have examined adverse events earlier in pregnancy. Although prenatal hypoxia induced by maternal smoking is a well-described schizophrenia RF^21^, there is no convincing study indicating any particular pregnancy stage is the most vulnerable. However, it is likely that maternal smoking may diminish once pregnancy is confirmed, suggesting that smoking induced prenatal hypoxia may be more common in the earlier stages of pregnancy.

With respect to influenza, the risk of schizophrenia is increased sevenfold if the mother was exposed in the first trimester with no increase at later gestational ages, however, this was only statistically significant when the first half of pregnancy was considered^22^. Reproductive tract infections also increase the incidence of schizophrenia in offspring but only when infection occurs either at conception or in the first few weeks of pregnancy as infection at later stages did not increase risk^23^.

In respect to prenatal stress, again it would appear that earlier stages of pregnancy are the most relevant stages. Women who were pregnant in the first trimester during either the German invasion of the Netherlands in 1940^24^ or the Arab–Israeli war of 1967^25^ had increased risk of producing children who developed schizophrenia compared to the other trimesters. A much larger study was reported recently by Khashan and colleagues using the national registry in Denmark. This study showed the death of one or more of the mother’s first degree relatives was linked to a statistically significant excess of schizophrenia, with a relative risk = 1.6–1.7, if the death occurred in the first trimester but not if the death occurred in the third trimester^26^.

Finally, nutrition deficiencies if present are unlikely to vary across pregnancy making it difficult to assess any particular stage. Nevertheless, in a limited number of studies where nutrition and stage of pregnancy were recorded, it would appear that trimester risk varies with each nutrient. For instance, low levels of maternal vitamin A during the second but not the third trimester is associated with a threefold increase in the risk of schizophrenia^27^. Another study has shown elevated levels of maternal homocysteine during the third trimester were associated with increased schizophrenia in adulthood^28^.

As nutrition is an easily modifiable variable the effects of supplementation on pregnancy outcomes can also be reported. Interestingly, the absence of folate supplementation during the first 10 weeks of pregnancy leads to childhood behavioural problems, such as social withdrawal, attention, and aggression at both 18 and 36 months in children^29,30^. Given the well-established link between low maternal folate and schizophrenia, it will be of interest to see if such children have a higher rate of progression to schizophrenia as such studies mature. However, all nutrient supplements are not necessarily benign, with one report of Omega-3 fatty acid supplementation during pregnancy leading to an increased risk of schizophrenia and attention deficit hyperactivity disorder symptoms, but only if taken during the initial phases of pregnancy^31^.

In summary, although not universally found, it would appear that adverse exposures exert a greater effect when experienced during the earlier stages of pregnancy. Such effects may be more profound as they are perhaps affecting more fundamental neurodevelopmental events such as cell proliferation and differentiation in the brain. This is likely to alter later events in brain ontogeny such as cell migration, axon migration, target selection, and synapse maturation. Here, it is important to consider the ontogeny of neuronal development in the human brain. Neurons with the capacity to synthesise dopamine (DA) are established very early in development, at approximately five weeks post-conception^32–34^. Whilst the contribution of cortical GABAergic and glutamatergic neurons to schizophrenia is undeniable these neurons are born at later stages of pregnancy. Given the centrality of DA dysfunction to schizophrenia, it is tempting to conclude that developing DA neurons may be particularly vulnerable to these adverse exposures. Indeed below, we will show data from animal models that appear to support this proposition.

## How do validated genomic variants in schizophrenia affect brain development?

Schizophrenia is clearly familial. The heritability of schizophrenia is estimated to be as high as (80%)^35^. Early studies on small, genetically homogenous populations produced findings suggesting single nucleotide polymorphisms, copy errors or splicing variants were increased in genes that could be plausibly associated with schizophrenia. These became known as “candidate” gene studies. Perhaps the most famous of these candidates was a translocation error in Disrupted in Schizophrenia 1 (DISC1). Much interest was generated regarding this gene due to its numerous functions in neuronal development^36^. While much has been learnt regarding the role of “candidate” genes in brain development and function, follow up studies in broader patient populations invariably failed to replicate any such association. Following many such false dawns with other such “candidates” the field had to recognise that schizophrenia was a polygenetic disorder of multiple variants of small effect.

Any search for potentially causal variants, therefore, would need very large sample sizes on a scale that was previously unimaginable. This necessitated the emergence of consortium-based GWAS studies. The current schizophrenia GWAS^37^ has largely ruled out coding variants within exons of previously established candidate genes. However there is variance near plausible genes, most prominently the L-type calcium channel CACNA1C and the dopamine D2 receptor, however, this is the exception rather than the rule. By default, this suggests that genetic variance for schizophrenia must reside within regulatory sequences. With the polygenicity of complex disorders such as schizophrenia now confirmed, we now see studies employing polygenic risk scores. Whilst still only accounting for as little as 7% of the total genetic variance of the disease^6^, these scores have a modest degree of predictive power in respect to patient diagnosis^38^.

One important observation emerging from such studies is the realisation that many of these common variants are involved in early development^39^. In particular, when considering the foetal brain, studies using the Human Brain Transcriptome microarray or the BrainSpan RNA sequencing data show that rare coding variants implicated by existing GWAS studies are associated with genes that are preferentially foetally expressed^40,41^. Another study has confirmed that genes associated with schizophrenia have greater transcriptional activity in foetal life^42^. Another study from the same group using more advanced sequencing technologies confirmed this relationship^43^. Thus one legacy of capturing the vast genetic variance of schizophrenia has been to alert the research community that disease polygenicity must be considered within an early developmental context.

Finally, one group has now used deep sequencing technology to assess gene transcript variants induced by gene splicing and shown that the isoforms of commonly expressed genes in brain change from foetal to adult life. Importantly it would appear that the isoforms expressed in the adult brains of patients with schizophrenia more closely resemble the foetal state^44^. The genes with these isoform shifts were significantly enriched for neurodevelopmental and cellular signalling processes.

## Investigating interactions between genomic variance and the developmental environment in schizophrenia; genome-wide environmental association studies

Historically G × E type interactions were explored using “genetic high-risk” cohorts (offspring of parents with schizophrenia). Despite some early associations linking a dramatic increase in birth complications in high risk-children who developed a psychiatric condition^45^ this was not replicated and remains a complex and sometimes controversial field (for a review see ref. ^10^). With the ever-increasing drive to integrate the known polygenicity of schizophrenia with the developmental risk, we are now entering the era of genome-wide environment interaction studies.

In perhaps the best of such studies, a PGRS was constructed from known alleles previously shown to have a significant (*p* < 5 × 10^−8^) association with schizophrenia from four individual case:control cohorts. When known, adverse early life pre- or perinatal complications were present, the PGRS was highly significantly associated with caseness however in the absence of early life complications there was no association^46^. Furthermore, the statistical significance of this association increased with severity of early life complications. Finally, the GWAS significant loci that interacted with birth complications related to genes that were highly expressed in placenta indicating this may be a major site for the genetic contribution of GWAS variants. Another study using an ethnically homogenous Danish population examined genome-wide association for schizophrenia with maternal cytomegalovirus infection. Although no variants were shown to reach the prior genome-wide significance threshold, two of the top three candidates cadherin-13 and *ZEB-1* have known roles in brain development^47^.

In respect to early life stress, a very recent study examined whether polygenetic risk scores interacted with early-life adversity in a genetically homogenous population to increase total risk^48^. This was essentially a pilot study in a smaller population to confirm feasibility. Whilst the authors confirmed that as expected, the PGRS predicted psychotic disorder and the cumulative effect of childhood adversity contributed to case prediction there was no additive effect, nor any interaction. Confirmation of this initial negative association between early life stress and genomic vulnerability will only come from planned much larger studies.

Another approach has been to examine if there was significant overlap between gene sets enriched for a particular RF and the GWAS variants implicated in schizophrenia. This has recently been done for genes related to ischaemia/hypoxia and the existing GWAS consortia data for schizophrenia and a significant enrichment was shown^49^. Variants within genes associated with schizophrenia interacting with ischaemia–hypoxia provide a specific starting point for functional and genomic studies related to obstetric complications. The GWEIS approach is still a long way from being able to provide aetiological targets in brain development. However, such approaches are providing leads for future functional interactions between developmental RFs and known genomic variance in schizophrenia.

## What role do epigenetic factors play in schizophrenia and could this affect brain development?

As previously mentioned most genetic variance in schizophrenia is in non-coding, potentially regulatory areas of the genome. In general, during development the more well-studied epigenetic processes such as DNA methylation and histone acetylation control the availability of the local chromatin environment for transcription and along with small non-coding RNA species help cells to maintain a differentiated state^50,51^. Here, I will discuss the epigenetic factors implicated in schizophrenia which may also have implications for developing brains.

DNA methylation is one of the most stable and best-characterised epigenetic processes in brain^52,53^. Cytosine residues within DNA are methylated on the fifth position in the cytosine ring. About 75% of DNA methylation largely occurs at CpG dinucleotides though in the brain it is also common to see other bases also methylated^54,55^. In the embryo, the DNA methyltransferase 3a and b enzymes are responsible for de novo DNA methylation and therefore most likely to be affected by developmental RFs. Methyl groups can also be oxidised by 10–11 translocation enzymes creating 5-hydroxymethylcytosine as an initial step in erasing methylation marks. These enzymes are highly expressed in neural progenitors but their expression drops dramatically when neurons differentiate^56^. Once stable methylation “marks” are placed on the DNA, various methyl-binding proteins are engaged in attracting histone deacetylation enzymes to further render the chromatin in a transcriptionally repressed state^57^.

In general, the genome becomes progressively more methylated with age. This has been recently confirmed for genes related to brain development, i.e. neuron differentiation and axonogenesis in the human prefrontal cortex (PFC)^58^. However more importantly when these authors examined CpG sites within schizophrenia GWAS-implicated variants^37^, this CpGs were more highly methylated and this finding was driven by sites that were more heavily methylated in foetal compared with adult life^58^. Importantly, reanalysis of such methylation patterns in adult PFC from patients with schizophrenia showed no such association. This work was largely replicated by a separate group that found the GWAS-significant loci were more than four times likely to be methylated in the foetal brain compared to regions unrelated to schizophrenia^59^.

The methylation and acetylation of histone residues is another major regulatory process governing transcription. DNA is wrapped around histone octamers containing two copies each of the histone variants H2A, H2B, H3 and H4 forming a chromatin structure^60^. Lysine and arginine residues which constitute the amino acids on the ‘histone tails’ are differentially methylated and acetylated to regulate transcription. Various different classes of enzymes Polycomb repressive proteins and the Trithorax activating proteins, histone acetylation and deacetylation enzymes are responsible for regulating chromatin stability. Developmentally, these enzymes “lock-in” the differentiation state of the cell.

A recent analysis of the pathways responsible for genomic regulation in schizophrenia, bipolar disorder and depression showed H3/H4 methylation and general histone and lysine methylation to be the top pathways implicated^61^. In addition, a recent study examined the acetylation of a particular histone lysine group (H3K27). H3K27 acetylation is a marker for chromatin relaxation in brain and varies with developmental stage. This study showed that in PFC, GWAS-validated variants were strongly enriched for H3K27 acetylation during foetal life but not in adult tissue^62^. This suggests that the regulation of GWAS-validated variants may be relaxed during brain development.

Finally, the role of microRNAs (miRNAs) in brain development and schizophrenia has to be considered. miRNAs are a class of small non-coding RNAs. They are ∼18–25 nucleotides long and are largely involved in gene silencing via their binding within their silencing complex and its recruitment to complementary sequences within target genes^63^. This leads to either the degradation of the target mRNA and or the repression of translation. Via such processes, miRNAs modulate cell fate, cell migration and cell polarisation during embryonic and early postnatal brain development as well as synapse development and the correct formation of neuronal circuits^64^. Alterations in the miRNA-137 locus were one of the first gene alterations to reach genome-wide significance in schizophrenia^65^. This finding was replicated in the later GWAS^37^. The risk-associated allele is reported to increase miRNA-137 expression. Overexpression of miRNA-137 impairs synaptic transmission in mouse hippocampal cells by reducing synapse formation^66^. Accordingly, the overexpression of miRNA-137 induces behavioural deficits of relevance to the disorder including sensory-motor gating deficits, impaired sociability, and cognitive deficits^67^. We recently showed miRNA-137 expression increases with embryonic age in both the rat midbrain and forebrain^68^. However, studies examining miRNA-137 and its interactions with the known gene variants in schizophrenia within the context of brain development have yet to be conducted.

Therefore, in summary, DNA methylation, histone acetylation/methylation and available miRNA data all suggest important interactions with known genetic variance in schizophrenia. In particular, all studies summarised here indicate this interaction is most relevant in the developing brain.

## How do epidemiologically validated RFs for schizophrenia affect the developing brain? Evidence from animal models

By necessity, animal models need to control the duration and severity of exposure within relatively genetically homogenous animals to reduce experimental variation. This means we can not assess the diverse life experiences/genetic backgrounds that collectively contribute to a psychiatric disorder. Despite this limitation, animal models are beginning to highlight convergent developmental pathways to disease. It is not our purpose here to provide an exhaustive listing of all findings from such models. For this, we refer the reader to a previous excellent review^69^. Rather our focus here is to (a) briefly describe how the developmental RF is modelled; (b) discuss whether there are critical vulnerable windows across gestation, and how these may affect developing and adult brain structure and function; (c) describe whether there are epigenetic alterations in developing brains and (d) outline the perceived mechanism/s.

### Animal models of obstetric complications/hypoxia: impact on the developing brain?

Pre-natal hypoxia can be modelled in terms of severity (8–10% oxygen) and duration using specially designed chambers^70^. Acute perinatal hypoxia is mimicked by brief exposure of rat newborns to 100% nitrogen atmosphere, whereas perinatal hypoxia-induced by prolonged labour has been modelled by caesarean section followed by immersion of the uterus in a water bath for a prescribed time^71^. Placental insufficiency or pre-eclampsia have been modelled in guinea pigs by inducing intrauterine artery restriction to reduce nutrient flow to the placenta^72^. Such models also induced widespread growth restriction and have now fallen out of favour.

Both pre- and peri-natal hypoxia models produce impairments in pre-pulse inhibition, social behaviours and cognitive deficits i.e. working memory and consistent with other developmental RF models, sensitivity to DA-releasing agents. They also appear to produce generalised cellular loss across the brain, diminished neuronal branching, alterations in DA release in response to psychomimetics, increases in tyrosine hydroxylase the rate-limiting enzyme in DA synthesis and other indices of DA dysfunction (for a summary see Meyer and Feldon)^69^. Currently, there is a paucity of data on embryonic brains subjected to pre- or peri-natal hypoxia.

Peri-natal hypoxia induces widespread epigenetic alterations in DNA methylation, chromatin methylation and acetylation [via hypoxia-inducible factor (HIF-1a)]. Additionally, a variety of miRNAs have all been shown to mediate hypoxia’s adverse effects in the developing brain leading to long-term alterations in transcription in important molecules in brain development and function. For a comprehensive review of the epigenetic alterations induced by hypoxia in developing brains see Bustelo et al.^73^. Severe hypoxia induces widespread cell death in the brain and is therefore unlikely to be highly informative for schizophrenias^74^. In contrast, more benign pre- or perinatal hypoxia activates both maternal HPA and inflammatory immune pathways so are likely to adversely affect the trajectory of multiple cellular systems in the brain^75^. Developing DA neurons, in particular, would appear to be susceptible with prenatal hypoxia reducing D1 and D2 receptor densities and the DA transporter in the striatum of foetal sheep^76^, as well as reducing DA and DA turnover in the brains of foetal guinea pigs^77^.

### Animal models of maternal immune activation: impact on the developing brain?

A wide variety of infections agents and chemicals have been used to induce maternal immune activation (MIA) in animal models. The two most prominently investigated are those using either the bacterial endotoxin lipopolysaccharide and the more popular synthetic double-stranded RNA analogue polyriboinosinic–polyribocytidylic acid (poly[I:C]) which mimic exposures to bacterial or viral infections respectively. These models have been thoroughly reviewed elsewhere^78^. These agents allow discrete control of dose and timing of exposure allowing the study of critical developmental windows and exposure thresholds. This is far more difficult to achieve with live infectious agents.

MIA with poly(I:C) induce abnormalities in brain structural and behavioural phenotypes, with earlier exposures i.e. gestational day (GD)9 in mouse leading to more pronounced abnormalities particularly in developing DA systems^79,80^. At a behavioural level, GD9 exposures produce offspring with hypersensitivity to DA-releasing agents, hyperlocomotion and impaired PPI. Later exposure, i.e., GD17 tends to affect the later developing cortical systems, most notably GABAergic neurons^79^. In general, GD17 exposure tends to affect cognitive processes such as reversal learning^79,81^. Also, the timing of exposure affects important structural brain abnormalities prominent in schizophrenia such as ventriculomegaly, with only the earlier exposure producing this abnormality^82^. This has not been as systematically examined in LPS models however in general the pattern would appear to be same^79,81^.

In terms of epigenetic alterations, the genome-wide methylation profile of PFC tissue has been analysed in neonates and mature offspring from poly(I:C) exposed dams at both GD9 and GD17 exposures. Essentially, MIA with poly(I:C) at GD17 induced differential methylation of genes involved in GABA neuron differentiation^83^. This is consistent with the GABA related abnormalities reported in the GD17 model^84^. In contrast, although there was substantial overlap between loci that were hyper- or hypo-methylated at both ages, MIA at GD9 induced methylation abnormalities in genes involved in very early processes in neuronal differentiation such as the WNT pathways with corresponding transcriptional changes^83^. In another study poly(I:C) at GD9 led to hypomethylation of the Methyl CpG-binding protein2 (Mecp2) promoter^85,86^. Mecp2 regulates gene methylation and therefore silencing via the recruitment of transcription factors dependent on the methylation state of the DNA. More importantly, Mecp2 has been shown to directly regulate DA neuron development^87^. Hypomethylation of this gene could, therefore, lead to inappropriate differentiation of developing DA neurons. Indeed poly(I:C) exposure at GD9 induces direct disturbances in the expression of specification factors specific for developing DA neurons^80^ and delays the differentiation and positioning of DA neurons in the developing mesencephalon^88^.

The molecules most implicated in the adverse effects of MIA on brain ontogeny involve the acute production of inflammatory cytokines, most notably (TNFα, IL-1β and interferon γ)^89^, IL-6^90^ and IL-17^91^. These cytokines affect the differentiation and maturation of neuronal circuits with molecules such as IL-1β directly mediating mesencephalic progenitor differentiation into DA neurons^92^ and TNFα and IL-6 concentration-dependently regulating DA neuron survival^89^. However, there are also interactions with stress pathways. An immediate and well-known consequence of MIA is the activation of the maternal HPA axis^93^. Recently it was shown such effects are long-lasting with low dose poly (I:C) exposure at GD9 rendering the adolescent brain more vulnerable to stress^94^. Enhanced microglial activation in hippocampus and PFC correlated with these behavioural effects.

### Animal models of maternal stress: impact on the developing brain?

Pre-natal stress is commonly modelled in rodents via maternal restraint, exposure to variable chronic unpredictable stress or exposure to glucocorticoids. Early postnatal stress is also modelled via a technique called maternal separation in which pups are removed from the nest for a prescribed period of time leading to minor thermal and hydration shock. Similar to maternal infection and hypoxia, maternal stress can also be titrated across gestation meaning critical exposure windows can be assessed. However, most researchers examine the effects of stress at either late gestational or in early postnatal life. Currently there is a paucity of data on the effects of prenatal stress within embryonic brains. In common with all previously discussed maternal exposures, animal models of prenatal stress produce behavioural sensitivity in adult offspring to DA-releasing agents. The nature of the stressor is important for schizophrenia-like phenotypes as variable stress leads to PPI impairments^95^, whereas restraint stress does not^96^. However, maternal restraint/unpredictable stress/corticosteroid exposure all reliably impair social and exploratory behaviours mimicking negative symptom phenotypes. Such exposures also impair cognitive function (reference and working memory) and reversal learning. For a summary of behavioural phenotypes of relevance to schizophrenia induced by maternal stress please see ref. ^97^.

11-hydroxysteroid dehydrogenase (Hsd11b2) is an important enzyme mediating the effects of maternal stress in the foetus. Hsd11b2 drives the interconversion of glucocorticoids and the inert 11-keto form (11-dehydrocorticosterone) in the placenta and foetal brain in response to chronic maternal stress. Chronic restraint stress during GD14–20 reduces Hsd11b2 mRNA, which was matched with increased DNA methylation of the Hsd11b2 gene promoter in placenta^98^. Also, pups exposed to stressed caretaker dams have persistent decreases in the crucial neurotrophic factor, brain-derived neurotrophic factor (BDNF). The persistent decrease in BDNF in the adult PFC is accompanied by increased BDNF promoter methylation. Importantly exposure to methylation inhibitors reverses these effects and normalised BDNF expression^99^.

The way maternal rodents groom their newborn pups is a proxy markers of maternal stress^100^. Meaney and colleagues have shown that grooming related to maternal stress induces a high level of the glucocorticoid receptor (GR) promoter methylation and as a result diminished GR expression in the hippocampus and impaired HPA function in these animals as adults^101^. Histone acetylation mechanisms were also proposed in this same study particularly regarding a region coding for NGF1-A (a transcription factor for GR). The translational relevance of this was demonstrated by Oberlander et al.^102^ who showed depression/anxiety in mothers was associated with an increased methylation at a predicted NGFI-A binding site with a corresponding increase in salivary cortisol stress responses in 3-month-old infants. The mechanism would likely be impaired GR expression in the infant’s brain.

The acute elevation in corticosterone induced by pre- and perinatal stress in animals is likely to have profound effects on brain development. Corticosteroids belong to the super-family of neurosteroids, such as the androgens, progesterone, etc., which have well-defined roles in brain development including governing neuronal proliferation, differentiation and as they are also transcription factors, gene expression. Not surprisingly an elevation in foetal cortisone is likely to affect the delicate balance of such steroids in normal brain development across the period where nuclear steroid receptors are first emerging in the embryonic brain^103^. As a transcription factor, it is likely corticosterone will recruit multiple proteins that are sensitive to epigenetic modifications^104^. In adults, stress steroids, in general, suppress immune response^93^. However, this may not operate the same in pregnancy with prenatal maternal stress increasing IL-1β in the placenta and foetal brain in utero^105^.

### Animal models of maternal nutritional deficiencies: impact on the developing brain?

Maternal deficiencies in protein intake, PUFAs, folate, iron and vitamin D can be easily modelled via specific dietary manipulations. This allows both examination of critical thresholds of deficiency, as well as supplementation, to examine neuroprotective potential. I have previously summarised the behavioural and brain structural phenotypes of relevance to schizophrenia produced by animal models of all these nutrient deficiencies^20^. In general, these models produce offspring with PPI deficits, hypersensitivity to DA releasing drugs and NMDA-antagonists reflecting positive symptom phenotypes. Less common, but also reported are deficits in exploration or social interaction reflecting the negative symptom phenotypes and cognitive deficits in domains such as working and reference memory. Maternal deficiencies in such nutrients also frequently produce structural brain abnormalities i.e. ventriculomegaly and alterations in hippocampal size and function, i.e., long-term potentiation. Also, supplementation with these nutrients frequently reverses these phenotypes as well as blocks their emergence in inflammation/stress/hypoxia models.

The effect of nutritional deficits at various stages of pregnancy in animal models has not been well explored. Perhaps this is not so surprising given the combination of short gestation times for rodents in combination with the long washout/wash in effects of dietary interventions compared with acute manipulations such as inflammation/stress/hypoxia. Although there are dramatic, potentially life-threatening outcomes, i.e., the absence of folate prior to neural tube closure, these are the exception rather than the rule. One study to date has examined the addition or removal of maternal dietary vitamin D across gestation. Adding vitamin D to developmentally vitamin D (DVD)-deficient rat dams at conception normalises vitamin D levels between GD7-14. This study showed that if vitamin D levels were normalised within the first 14 days of pregnancy this prevented the well-described locomotor sensitivity to NMDA-antagonists in this model^106^.

Although the exact developmental pathways targeted by these nutritional deficiencies leading to schizophrenia are unknown, they all affect epigenetic processes in developing brains. For instance, iron is a vital co-factor for enzymes involved in histone or DNA methylation^107^. Other factors such as folate can act as direct methyl donors for these same methylating enzymes^107^. PUFA deficiency during embryonic development in mice leads to hypermethylation of the promoters of the important developmental nuclear receptors retinoid X receptor (Rxr) and peroxisome proliferator-activated receptor (Ppar) in brain delaying differentiation^108^. Folate and PUFA supplementation also regulate DNA methylation in the placenta^109^. In respect to general maternal nutrition, reduced energy intake decreased DNA methyltransferase activity and altered histone methylation and acetylation and reduced methylation of the foetal hypothalamic proopiomelanocortin promoter and GR promoter in foetal sheep^110^. A 50% reduction in maternal protein intake also leads to long-term hypermethylation in the majority of genes within the hippocampus and PFC including important developmental genes such as Mecp2^111^. Although the targeting of epigenetic processes by DVD-deficiency in developing brains is highly likely given vitamin D’s role in numerous epigenetic processes^112^ no so such studies have yet emerged.

Common early molecular features emerging from the non-nutritional RF models are that developing brains are exposed to excessive corticosteroids as a result of impaired maternal HPA function as well as increased exposure to inflammatory factors. Although deficits in maternal nutrition have been linked with altered HPA function in adult offspring^113^, whether the developing brain is exposed to increased levels of these agents has not been well examined. DVD-deficient dams have increased maternal corticosterone production in response to restraint stress^106^ and also an increased placental inflammatory response^114^. Maternal iron deficiency elevates placental inflammation^115^ as well as corticosterone production in both the dam and newborn brain with corresponding alterations in GR expression in the developing hippocampus^116^. Maternal inflammation also frequently leads to acute iron deficiencies^117^. In addition, maternal protein deprivation is a well-known regulator of HPA function^113^. Maternal PUFA deficiencies exacerbate inflammatory cytokine production in the placenta and foetal brain in response to an inflammatory agent^118^. Although maternal PUFA deficiencies exacerbate HPA response to stress in adult offspring, the effects of PUFA deficiency on corticosterone exposure in the developing brain have not yet been studied^119^. Finally, although prenatal folate supplementation reverses the effects of early postnatal stress^120^ and suppresses maternal immune response^121^, to the best of our knowledge, whether its dietary absence increases foetal brain exposure to corticosterone or inflammatory factors has not been reported.

## Epidemiologically validated RFs: convergent processes affecting brain development?

The developmental animal models discussed here all produce robust behavioural phenotypes of relevance to the positive–negative and cognitive symptoms of the disease in adult animals. These overlapping phenotypes suggest an early common pathogenic mechanism/s. Unfortunately, apart from a concentrated effort to understand early brain changes in the MIA and DVD-deficient models, there has been far less focus on the developing brain in the other models. However, certain themes are emerging.

### Most models increase foetal brain exposure to glucocorticoids and/or inflammatory factors

**Fig. 2:**
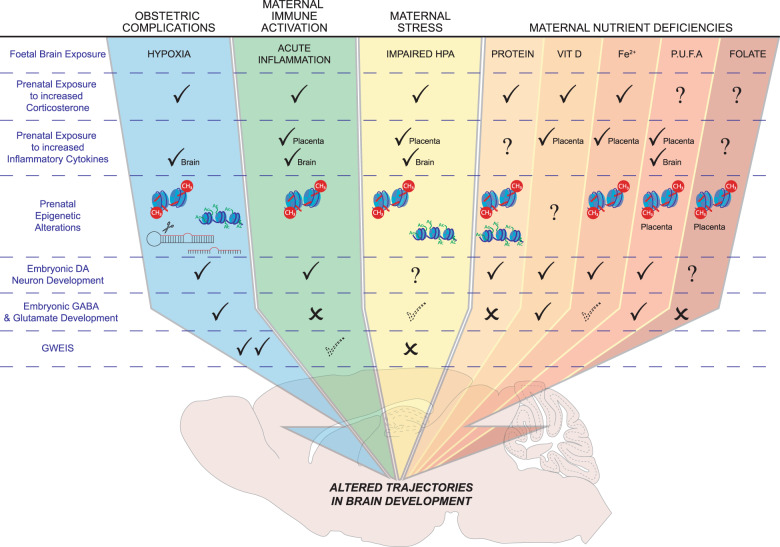
Epidemiologically-validated developmental risk factors for schizophrenia and the convergent processes affecting brain development. This figure depicts the multiple convergent processes operating across the prominent developmental risk factors for schizophrenia. Almost all exposures increase foetal brain/placental exposure to increased glucocorticoids and inflammatory factors. There are also multiple alterations to early epigenetic factors governing gene expression, with most risk exposures affecting DNA methylation, however certain exposures also affect histone acetylation/methylation and micro RNA production. At a cellular level a surprisingly high number of exposures affect the early ontogeny of dopamine neurons. Effects on the development of other brain cell types i.e. GABA or glutamateric are also affected but reports of alterations to these neurons are far less frequent. Also noted is whether genome-wide environmental interaction (GWEIS) studies have been conducted for these developmental risk exposures. Abbreviations: DA, dopamine; GWEIS, genome wide environment interaction study; HPA, hypo-thalamic pituitary axis; Vit D, vitamin D; Fe^2+^, iron; PUFA, polyunsaturated fatty acids.  Increased exposure validated in developing brain/placenta.  Weak evidence. X No effect. ? Not studied.  Altered DNA methylation.  Altered Histone acetylation/methylation.  Increased micro-RNA.

To some extent, all of these RFs can also be considered prenatal stressors. As such, an examination of the scant literature in this domain reveals that all non-nutritional and most nutritional models induce maternal glucocorticoid secretion and exacerbate inflammatory cytokine production either in the foetal brain or placenta (Fig. 2). The crosstalk between these two major signalling pathways has long been known^93^. Glucocorticoids and inflammatory cytokines have well-described impacts on developing neurons. Corticosterone is an important developmental nuclear steroid that directly regulates transcription for myriad genes relevant to the developing brain^122^. Inflammatory cytokines are central for neurogenesis, early neuronal specification, gliogenesis and their effects on radial glia can affect early neuronal positioning^123^. It is, therefore, highly likely that these exposures will combine to change early cellular differentiation/specification/migration and axonal and synaptic connectivity in developing brains. A re-examination of these very early events in shaping brain ontogeny and in particular determining whether certain cells/circuits are primarily targeted by early-life exposure to glucocorticoids or inflammatory factors is now required.

### Most models produce early and possible persistent alterations in diverse gene silencing mechanisms?

As outlined above the genome in patients with schizophrenia is differentially methylated particularly in genomic regions important for foetal development^58,59^. Histone and lysine methylation pathways are also implicated across multiple psychiatric conditions^61^ and changes in these factors would appear to be primarily relevant during brain development^62^. Alterations in miRNAs particularly miRNA137 is one of the most replicated genetic abnormalities in schizophrenia^37^ and embryonic miRNA regulation in developing rat brains would appear to be highly dynamic^68^. In re-evaluating the animal modelling literature another striking convergent feature is alterations to these same epigenetic processes in either the developing brain or placenta. There is even likely to be some crosstalk between de novo gene methylation and foetal brain corticosteroids with data only now emerging showing corticosterone recruits the glucocorticoid receptor (GR) onto promoters of DNMT1^124^.

### Most models produce early alterations in developing DA systems

A remarkably consistent and critically important feature of all animal models that reflect pre- and perinatal risk factors for schizophrenia (both nutritional and non-nutritional) is that they all appear to produce adult offspring with a heightened behavioural sensitivity to the psychomimetics amphetamine or cocaine. Amphetamine acts primarily through enhancing DA release, and cocaine acts by blocking DA reuptake. The evidence for DA abnormalities in schizophrenia represents the most long-standing hypothesis for this disorder^125^ and continues to be bolstered by clinical positron emission tomography studies^126^.

Along with the DA hypothesis, the neurodevelopmental hypothesis of schizophrenia probably represents the next most long-standing hypothesis regarding the origins of this disorder^1^. Previously I speculated on whether all adverse prenatal exposures change the ontogeny of DA systems, thus bringing these two long-standing ideas together into the one hypothesis^127^. This proposal was based on limited data from the MIA and DVD-deficiency models. However, this idea has since been considerably bolstered by further data in these same models over the last eight years. Space precludes a detailed summary of these new findings, however in summary, maternal exposure to both RFs alter (a) the expression of factors important in the early specification of DA neurons such as Nurr1, Ptx3, and lmx1a^80,88,128–130^; (b) the enzymes involved in the production^88,128,130–132^ and breakdown (COMT/MAO) of DA along with corresponding changes in foetal brain DA levels^80,133,134^ (c) the early positioning of DA neurons within the foetal mesencephalon^88,130^. Finally, mechanisms governing the direct regulation of COMT^135^ and a receptor component cRET^136^ required for the essential dopaminergic neurotrophic factor, glial-derived neurotrophic factor, have now been described in the embryonic DVD-deficient brain. Thus, it would appear highly plausible that such a constellation of early alterations to the molecular and spatial architecture of developing DA neurons would lead to alterations in DA function in more mature brains, potentially explaining the origins of DA signalling disorders produced by these models.

There has been far less focus on developing brain circuits in the other models, however, there are suggestions from the scant data available that developing DA circuits may be similarly vulnerable. For instance, chronic hypoxia alters DA turnover and DAT expression in foetal sheep and guinea pig brains^76,77^. When postnatal [(P) (day 14)], rat pups from a model of maternal under-nutrition were examined, they were shown to have reduced DA1 receptor density across the hypothalamus and increased DA2 receptor density in the arcuate nucleus. Unfortunately, pups were not studied at any earlier developmental ages, nor was any other brain region examined. In a model of maternal protein deficiency, early changes in potassium-evoked DA release from hippocampal slices was shown in P15 rat pups and this early functional abnormality persisted into adulthood. Rat pups (P10) from dams with moderate anaemia (10–20% iron deficiency) have a dramatic increase in brain DA. This increase persisted into weaning but was less dramatic. There were also increases in DAT expression, and regional changes in DA2 receptors correlating with very early sensorimotor defects. Rat pups from maternally iron-deficient dams also have early reductions in bar gripping and forelimb placement which persist into weaning^137^. A number of early studies have shown that dietary omega-3 fatty acid deficiencies produce alterations in both brain DA content and DA receptor expression in adult animals To the best of our knowledge, there has been only one study that varied the fatty acid content of the maternal diet and showed that low levels of phosphatidylserine and phosphatidylethanolamine led to higher levels of DA in the brains of newborns.

### Some models produce early alterations in GABA/glutamatergic systems

Many of these developmental RFs also affect GABA and glutamate function in adult offspring. However, there is minimal data on how these RFs affect the early development of these neurotransmitter systems. Perhaps the best evidence comes from studies of prenatal hypoxia. Prenatal hypoxia alters embryonic cortical GABA cell number and positioning up to P2 in the cortex but this reverses from P4^138^. Prenatal hypoxia at GD17 in mice also induces an immediate reduction in foetal cerebral cortex levels of the GABA synthesising enzyme, glutamate decarboxylase^139^. Prenatal hypoxia also upregulates the NR1 subunit of the NMDA receptor for up to 3 days post exposure in white matter microglia but this is reversed from 7 days post exposure^140^.

There is no evidence of effects of MIA on glutamate and GABA systems in the developing brain. Effects on adult brain however are clearly dependent on the timing of exposure. While MIA has no long lasting effect on glutamate or GABA levels^141^, exposure at later gestational ages such as GD17 leads to alterations in GABA and NMDA NR1 subunit expression in juveniles and adult offspring^141–143^. Earlier gestational exposures, however, have little to no effect on this neurotransmitter systems^141^.

Despite consistent findings in adult offspring, there is also very little data on the effects of prenatal stress on developing GABA or NMDA systems. However, prenatal stress does induce delays in GABAergic progenitor migration and this appeared related to persistent alterations in microglial activation^144^.

In respect to nutritional RFs, DVD-deficiency induces a small reduction in glutamate and GABA in the neonatal hippocampus but no other brain region. Interestingly there were widespread reductions in the levels of glutamine (12–24%) across the neonatal brain consistent with a possible imbalance in glutamate/GABA recycling^134^. The effects of prenatal iron deficiency on GABA and glutamate systems in embryonic brains is unknown. However, there is one report showing the activities of GABA and glutamate recycling enzymes (glutamate dehydrogenase, glutamate decarboxylase and GABA-transaminase) along with GABA content are decreased from P14 in rat brains^145^. Maternal deficiencies of PUFAs lead to small increases in GABA and reductions in glutamine in female neonatal rat brains^146^. There are no reports on the effects of folate or protein deficiencies on developing GABA or glutamatergic systems.

## Conclusion

In this review I have summarised the evidence showing that epidemiologically-validated developmental RFs for schizophrenia adversely affect brain development in experimental animals. Although not universally demonstrated it would appear that the earlier gestational windows may exacerbate this risk. In considering the current polygenetic contribution to schizophrenia it would also appear that risk variants are more likely to be expressed during development. Although it is very early days in examining the interaction between maternal RFs and polygenic risk, the GWEIS approach is providing initial data suggesting we may find future functional interactions between developmental RFs and known genomic variants in schizophrenia. In considering the role of epigenetic factors, it would also appear that the polygenic variants currently implicated in schizophrenia are likely to be differentially epigenetically regulated during development compared to adulthood.

One exciting development addressing this issue is the Integrative Psychiatric Research (iPSYCH) consortium^147^. This consortium exploits the excellent patient records from the Danish Civil Registration System coupled with the availability of DNA obtained from the Danish Neonatal Screening Biobank. Whole exome sequencing, methylation profiling, metabolome profiling, vitamin D and, inflammatory assessments will be made on this large population in the hope that this may reveal how genetic and adverse early developmental environments interact to cause psychiatric disorders.

Of course there could be additive effects between multiple developmental RF exposures in the progression towards schizophrenia. In the animal model literature, gene × RF environment interactions have been widely explored, but combinations of environment RF are less common. Even when these are modelled, they are almost exclusively pre- × postnatal RF exposures rather than combinations of prenatal RFs^148^. However, there are very provocative data from the clinical literature. A recent study showed an additive interaction between three non-maternal RFs, namely—cannabis use, childhood trauma and urbanicity in predicting future psychotic symptoms in the general population^149^. In addition, in a landmark study by Stepniak and colleagues, it was shown that whilst PGRS had no predictive power for the age of onset in a sample population of 750 males with schizophrenia, individuals who had been exposed to four or more environmental RFs (of which only one was prenatal, i.e., perinatal birth complications) had an age of onset that was nearly a decade sooner than those that had no adverse environmental exposures^150^. In a much smaller study of at-risk individuals, the authors showed combining pre- and postnatal non-genetic RFs for schizophrenia predicted conversion to psychosis^151^. Some authors are now even advocating for a polyenviromic risk score^151^.

In respect to the animal models of these RFs, I have summarised what I feel to be the convergent molecular and neuronal pathways that are targeted (for a summary see Fig. 2). The current data from such models suggest activation of early stress/inflammatory pathways in developing brains may be a factor in virtually all such exposures. Similarly, at a cellular level, there are convergent findings from most models that the developmental trajectory of DA neurons may vary widely from non-exposed animals. There is also some support for alterations in embryonic GABA and glutamatergic neurotransmitter systems but this is not convergent across all models. Although highly speculative, I propose that unless these very early molecular and structural alterations to neurotransmitter development are not rapidly compensated then the embryo would be non-viable. MIA and DVD-deficiency produce stark early reductions in essential specification factors for developing DA neurons such as Nurr-1, Ptx3, Lmx1a, TH, etc.^80,88,130,132^. Numerous studies have shown homozygous deletions of these factors are embryonic lethal^152–154^. It is illuminating that in both models, the early gestational reductions in these essential dopaminergic genes are rapidly reversed often leading to heightened expression compared to controls by birth^127^. The factors responsible for this over-compensation remain obscure. Although highly speculative, such extreme buffering/overcompensation may eventually create a pathogenic process that itself cannot be successfully buffered later in life potentially leading to psychiatric outcomes. I outline this speculative mechanism as option 4 in Fig. 1.

There remain large gaps in this literature and we need to design studies that are directly aimed at establishing whether the molecular and neuronal findings summarised here represent true points of early convergence in understanding schizophrenia aetiology. We also need more studies that assess the longitudinal maturational journey of all major cell types in the brains from such models. We still have very few preclinical studies that focus on the longitudinal aspects of developmental brain maturation postnatally and up to late adolescence after exposure to such developmental RFs. Such studies are important as they may help to uncover which pathogenic processes initiated early in brain development may illuminate targets for intervention. This becomes important given the recent focus on intervening during the prodromal stage of schizophrenia^155^. One reason for the dearth of longitudinal studies in this field may be a practical one, with the invasive molecular and cellular techniques required, demanding multiple cohorts. The hope is that from such studies we will begin to understand what processes are initiated to compensate for the earlier insults and indeed when such pathways decompensate^39^ leading to disease-related phenotypes.
